# Low protein diets in patients with chronic kidney disease: a bridge between mainstream and complementary-alternative medicines?

**DOI:** 10.1186/s12882-016-0275-x

**Published:** 2016-07-08

**Authors:** Giorgina Barbara Piccoli, Irene Capizzi, Federica Neve Vigotti, Filomena Leone, Claudia D’Alessandro, Domenica Giuffrida, Marta Nazha, Simona Roggero, Nicoletta Colombi, Giuseppe Mauro, Natascia Castelluccia, Adamasco Cupisti, Paolo Avagnina

**Affiliations:** Department of Clinical and Biological Sciences, SS Nephrology, ASOU san Luigi, University of Torino, Torino, Italy; Nephrologie, CH du Mans, Le Mans, France; Department of Surgery, SS Dietetics, città della salute e della scienza, University of Torino, Torino, Italy; Department of Experimental and Clinical Medicine, SCDU Nephrology, University of Pisa, Pisa, Italy; Department of Clinical and Biological Sciences and of Oncology, Library, ASOU san Luigi, University of Torino, Torino, Italy; Department of Clinical and Biological Sciences, SSD Clinical Nutrition, ASOU san Luigi, University of Torino, Torino, Italy

## Abstract

Dietary therapy represents an important tool in the management of chronic kidney disease (CKD), mainly through a balanced reduction of protein intake aimed at giving the remnant nephrons in damaged kidneys a “functional rest”. While dialysis, transplantation, and pharmacological therapies are usually seen as “high tech” medicine, non pharmacological interventions, including diets, are frequently considered lifestyle-complementary treatments. Diet is one of the oldest CKD treatments, and it is usually considered a part of “mainstream” management. In this narrative review we discuss how the lessons of complementary alternative medicines (CAMs) can be useful for the implementation and study of low-protein diets in CKD. While high tech medicine is mainly prescriptive, prescribing a “good” life-style change is usually not enough and comprehensive counselling is required; the empathic educational approach, on which CAMs are mainly, though not exclusively based, may support a successful personalized nutritional intervention.

There is no gold-standard, low-protein diet for all CKD patients: from among a relatively vast choice, the best compliance is probably obtained by personalization. This approach interferes with the traditional RCT-based analyses which are grounded upon an assumption of equal preference of treatments (ideally blinded). Whole system approaches and narrative medicine, that are widely used in the study of CAMs, may offer ways to integrate EBM and personalised medicine in the search for innovative solutions respecting individualization, but gaining sound data, such as with partially-randomised patient preference trials.

## Background

### Chronic kidney disease (CKD): an example of a highly complex, life-long disease

Dialysis and transplantation are life-long, life-sustaining treatments requiring multiple drug therapies, and a high technological level of “mainstream medicine” [1–3]. However, like all life-long, chronic diseases they require not only integration of complex therapies into everyday life (multiple drugs in the earlier phases of the diseases, and then drugs and dialysis or drugs and a kidney transplant, not necessarily in this order), but also a change in lifestyle, including diet.

In this regard, CKD treatments merge technology (dialysis, transplantation, multiple drug therapy), which belongs to/“mainstream medicine”, and lifestyle interventions (exercise, weight loss, low-protein diets) which are conversely often considered a part of the holistic approach attributed to “complementary and alternative medicine” (CAM).

While several comprehensive reviews have addressed the issue of the efficacy of various therapeutic approaches, including diet, to date, no reviews have been dedicated to the relationship between a mainstream and a CAM approach to low protein diets in CKD patients.

Indeed, nutritional treatments share these two facets: several recent papers aimed at a vast readership of family practitioners which assessed diverse diseases cite the addition of different foods or the use of specific diets as part of CAM approaches [4–7].

For at least a century chronic uraemia has been considered an example of “protein intoxication”. Thus, in the history of “renal medicine”, diets have been attempted as a way to correct the metabolic alterations of kidney failure as much as possible [8, 9]. Indeed, the first systematic studies on low-protein diets in uraemia patients started with the observation that a protein-restricted diet was effective in reducing the symptoms of “uraemic toxicity” and that it was even able to prolong life [10–12]. This role of life-prolonging therapies was maintained in western countries until dialysis became widely available; availability of dialysis, no more a problem in Europe, is however non universal on a global scale [12–21]. Reconsidering the goals and modalities of low-protein diets in progressive renal diseases and keeping in mind the lessons of CAMs may offer an interesting outlook in the context of “high-tech” CKD care [20, 22–24]. At a time in which growing interest (and business) in CAMs may divert attention from high-tech medicine, “playing in the two fields” may also aid the physicians in helping patients find effective coping strategies.

### Chronic kidney disease in the new millennium: why is it difficult to measure progression?

At the beginning of the new millennium, “renal insufficiency” was redefined and CKD became an umbrella term encompassing several affections having variable evolution rates towards end-stage kidney disease (ESRD) [25–27]. The staging of CKD underlines the similarities of all advanced kidney diseases, despite different progression rates which depend upon race, age and underlying nephropathy [25–35].

Therapies aimed at slowing CKD progression have to deal with high baseline heterogeneity that hinders the demonstration of their long-term advantages and drawbacks [30–36]. Even the method that is used to measure progression rate is a matter of controversy. Furthermore, differences in the hard outcomes, namely start of dialysis or death, require trials with long observation times which are expensive and are often affected by many confounding factors [28–36].

Several studies have focused on different therapeutic goals which may partially overlap: these goals include removing the cause of CKD, treating the metabolic derangements, correcting lifestyle habits that are not necessarily consequent to CKD but that may potentially worsen the diseases, and reducing the work-load on the remnant nephrons [20, 37, 38].

Dietary interventions in general, and low protein diets in particular may play a role at all these levels by, for example, treating obesity, correcting hypophosphataemia, avoiding processed foods, and most importantly, reducing the work-load on the remnant nephrons.

Furthermore, the outcome measure is non-univocal: slowing the progression of CKD is not synonymous of delaying dialysis [20].

Long-term follow-up of either homogeneous populations with similar baseline progression rates or of very large cohorts is ideally needed to demonstrate an effect of dietary interventions (and, indeed, one of the observations of the famous MDRD study was that it would have probably given different results over a longer follow-up [39–41]). The initial GFR decrease, achieved by “renal rest”, or correction of the relative hyperfiltration may interfere with short-term analyses [42, 43].

Conversely, delaying dialysis requires attaining stable metabolic balance that is, at least partly, independent of GFR. However, the analysis of this short term goal is only apparently easier since it is deeply entangled with the pharmacological “mainstream approach” and reflects various policies of dialysis start which are not merely based upon (relatively) simple measures, such as GFR. The new Canadian guidelines on dialysis start clearly underline that whenever an “intent to defer” policy is chosen, wise clinical judgement cannot be replaced by formulae [44].

Heterogeneity of diseases, heterogeneity of progression, and heterogeneity of goals and measures add to the charm, but also to the complexity of the analysis of dietary approaches in CKD patients. Without a change in the approach, the issue will probably remain open forever.

### Low-protein diets in CKD: why is the evidence not evident enough?

Many of the questions posed in the 1986 paper published in the Lancet entitled: “Dietary treatment for chronic renal failure, ten unanswered questions” are currently still unanswered. Among them, some highlight the complexity and heterogeneity of chronic kidney disease (Does chronic renal failure always progress?), while others underline the difficulties in standardization of low protein diets in clinical practice (When should a low-protein diet start? Which low-protein diet? How should we assess progression, nutritional status or compliance?) [45]. However, in the literature there are several evidence-based demonstrations of the possible role low protein diets may play in delaying ESRD, including several RCTs and a Cochrane review [46–58]. Furthermore, we are not aware of any large studies demonstrating a higher progression rate in non-malnourished, on-diet patients; in addition, data on lower costs are concordant [48, 49, 51], and there is no evidence showing that being on the diet results in any survival disadvantage after the start of dialysis [51, 59, 60].

The risk of malnutrition is frequently cited, but the data are at best as inconclusive as those cited by the diet “advocates” [61–68].

Low protein diets are however still underutilized [20].

Table 1 summarizes some of the reasons why even “best quality” evidence is often considered non conclusive or does not affect the clinical management of CKD patients. Many of these reasons underline how the prescriptive modality, which is typical of high-tech medicine, may be less suitable than the educational modality, which is typical of CAMs, for the management of diets in CKD patients.

**Table 1:** Some comments on the main RCTs con LPDs enrolling at least 50 patients per arm

Study	Comparators	N and groups	Main results	Limitations	Other comments
Klahr S et al., N Engl J Med. 1994 (MDRD) [39]	LPD vs No diet; LPD vs LPD	Study 1, 585 patients: usual diet: 1.3 g/Kg/day LPD: 0.58 Study 2, 255 patients on or vLPD (plus BP control)	moderate CKD: small benefit LPDs. severe CKD: no difference in ESRD progression on LPDs and vLPD	Highly complex study. The results are given as ITT; however PP analysis shows a significant effect of LPDs, thus highlighting the role of compliance.	The largest RCT on LPDs leading to inconclusive results: it may be also read as measure of the limitations of RCTs analysed as ITT, due to compliance issues
Brunori G, et al. Am J Kidney Dis. 2007 [53]	vLPDs versus dialysis in the elderly (stage 5)	56 patients in each group (296 screened)	vLPDs are effective in delaying the need for dialysis without increasing mortality	Only about 30 % of the initial population accepted being randomised. No information on the follow-up and outcomes of the excluded patients.	The only study randomizing dialysis vs vLPDs; highly relevant even if randomizing such intrusive issues may be perceived as “unethical”
Cianciaruso B, et al. Nephrol Dial Transplant 2008 [57]	0.55 LPD and 0.8 LPD in CKD stage 4–5	200 patients on 0.55 diet, 192 on 0.8 diet (screened 753; initial randomization: 423 pts)	LPD at 0.55 g/kg/day guarantees better metabolic control than a 0.8 diet.	Relatively low compliance in the 0.55 study group (compliant patients: 27 % in the 0.55- Group and 53 % in the 0.8-Group), thus blunting the conclusions. At present 0.8 should be a “normal protein”	Very large study, on two “moderately restricted LPDs: it shows that even within the “moderate restriction range” the lower the better, without risk of malnutrition
Garneata L et al. JASN 2016 [59]	vLPD vs LPD in CKD stages 4–5	vLPD: 104 patients LPD: 103 patients (Screened 1413; non compliance is the main reason for non being randomised)	Better correction of metabolic abnormalities and lower need for dialysis in the vLPD cohort.	Only 14 % of screened patients were randomized. Optimal compliance is a requisite for randomization, indirectly suggesting that these diets are an option for relatively few CKD patients.	The largest recent RCT targeted on supplemented vLPDs vs LPDs. Underlines the importance of vegan diet and of supplementation.

Legend: *LPD* low protein diet, *vLPD* very low protein diet, *CKD* chronic kidney disease, *BP* blood pressure; numbers indicate the prescribed protein intake per Kg per day

The first reason why the treatment with the best cost-benefit ratio is underutilized in the expensive CKD scenario may be the difference between the amount of time it takes to prescribe a drug or define the parameters of a dialysis treatment (mainly requiring technical skills), and to recommend lifestyle changes (especially in the case of diet) which are deeply rooted in personal preferences and social habits. In this regard, the barriers for the implementation of LPDs may be the result of the difficulty in conciliating the prescription and follow-up of a diet with a classic “mainstream medicine” approach. Some comments regarding the MDRD study, the largest randomised controlled study on LPDs in CKD, are in line with this interpretation: some experts claimed that MDRD actually demonstrated that prescribing a diet is not enough, since patients tend to lean to the dietary habits of their choice [39–43]. Hence, the results of the “intention to treat” analysis (diet as a “mainstream prescription”) are negative (no advantage of an LPD on CKD progression), while the results of the “per protocol” analysis (diet as an educational intervention) suggest a positive role for LPDs in slowing CKD progression (Table 1).

The outstanding results obtained in some centres with a great deal of experience in selecting and following-up patients on low protein diets have rarely been replicated in other settings [51–59]. The selection issue is fundamental: randomization is felt to be unethical on dialysis: this is why peritoneal dialysis and hemodialysis have never been randomised and their results are acknowledged as a matter of prescription and therapeutic adherence. Strangely enough, the same caveats are not applied to diets. Diet, as well as dialysis, depends upon the patients’ choices, and the few RCTs that were performed on dialysis frequency (3 versus 6 dialysis sessions per week) and those carried out on very low protein diets share an extremely low enrolment rate (in the 10–30 % range) [52–54, 59, 69–71]. Accordingly, one of the main criticisms of the unique Brunori study on diet versus dialysis is that it enrols about 30 % of the population screened for recruitment (and the percentage of cases randomised in the Garneata trial on very low protein diets is about 10 %) (Table 1). Such a low prevalence demonstrates the presence of a strong patient preference and suggests that adapting the prescription to the individual may be more successful than randomizing therapies. These issues merge with those related to quality of life, as will be further discussed [72–77].

### Diet is not a drug: the “low-protein menu” in CKD

Wikipedia, the world’s largest source of on-line information, states “diet is the sum of food consumed by a person or other organism”; however, in clinical practice, the word “diet” is often interpreted with a restrictive (or even punitive) connotation, meaning the reduction of at least some foods [77, 78]. Limiting, reducing, and abolishing, are probably not the best slogans for a “marketing strategy” to improve compliance among CKD patients. Indeed, some authors presently prefer the terms “nutritional treatments” or “nutritional interventions” to define strategies aimed at changing dietary habits and not at restricting the use of some (many-most) foods [77–80].

The concept of low-protein diets is presently undergoing a substantial change, mainly as a consequence of the changes in the definition of “adequate” protein intake which has shifted in recent years from 1–1.2 g/Kg/day to 0.8 g/Kg/day, according to the most recent FDA Reference Daily Intake or Recommended Daily Intake [81, 82]. The issue of dietary protein content in the overall population is complex, and is entangled with that of healthy lifestyle; the rediscovery of traditional and Mediterranean diets, and the attention to reducing additives and processed foods are two emerging aspects of this issue [83–90].

A low protein diet is not necessarily a healthy diet; however, the cultural trend towards reducing proteins and canned, preserved and processed foods plays in favour of our patients, for whom the older “moderate” protein restriction (0.8 g/Kg/day) is now synonymous of a “biologically normal/adequate” protein intake, and who may choose a vegan-vegetarian or traditional diet (for example “Mediterranean”) with a lower risk of being “discriminated” in a carnivorous world [77].

Table 2 shows the main “menus” of low protein diets as defined according to; the daily protein intake relative to body weight, to the main foods, to the room for personalization and to the type of approach (“mainstream prescription” versus “CAM education”).

**Table 2:** The “LPD menu”: some reflections on compliance

Type of diet	Protein restriction (g/Kg/bw)	Main features	“Best patients”	Main advantages	Main disadvantages	Personalization; main approach
“Traditional”	0.6–0.8 g/Kg/day; mixed proteins	Modulated upon quantity of usual food; in moderate and hot climates, traditional cuisine is more plant based, and returning to the roots may be useful	Mediterranean- Asian origin; careful with preparation, cook their own food	A very natural approach, adapted to all settings, doesn’t require special food,	Demanding: requires special attention to quantity and quality of food	Large room for personalization, discovery and rediscovery of traditional cuisine; flexible; Educational approach is needed.
Vegan	0.6–0.8 g/Kg/day; vegetable proteins	Unrestricted vegan diets are usually in the 0.7–0.9 g/Kg/day protein intake range; due to the different bioavailability, a 0.7 diet roughly corresponds to a 0.6 mixed protein diet	“New age”, young people who want to avoid supplements or special food; Cook their own food	A “trendy” approach, due to the diffusion of veganism in the western world; a natural diet that may have other favourable effects on health	Demanding: requires special attention to quality of food and to the integration of legumes and cereals. Risk of B12, vit D and iron deficits	Quite good room for personalization, especially for not becoming boring; relatively flexible; Educational approach is needed.
Vegan supplemented	0.6 g/Kg/day; vegetable proteins, supplemented with a mixture of amino- and keto-acids	Based upon forbidden (animal origin) and allowed (all other) food. Animal-derived food is allowed only in “free meals”	young working people, who want a simple diet, easily adapted to any situation	A simplified approach: supplements avoid the need to integrate legumes and cereals, thus reducing the risk of nutritional deficits	Adding pills to the usual, often already demanding drug list. Expensive where supplements are not supplied by the health care system	Some room for personalization, especially for not becoming boring; relatively flexible; Educational approach has to be combined with a prescription approach (supplements)
Protein-free food	0.6 g/Kg/day; mixed proteins	Protein-free pasta, bread and other carbohydrates	Mediterranean- Asian origin; elderly people who do not want to change their habits	May allow a reduction of proteins without changing eating habits	The protein-free food tastes different and may not be “tasty”, it is expensive where foods are not supplied by the health care system. The food has to be prepared separately	Large room for personalization, may preserve previous habits in Mediterranean settings; relatively flexible; Prescription approach for protein-free food.
Very low-protein supplemented (with or without protein-free food)	0.3 g/Kg/day; vegetable proteins, supplemented with a mixture of amino- and keto-acids; higher dose as with the 0.6 diet	Based upon forbidden (animal origin) and allowed (all other) food. Animal-derived food is allowed only in “free meals” (usually no more than 1 per week)	Highly motivated patients who do not want to start dialysis or are waiting for transplantation	The most effective approach for delaying dialysis start	Adding many pills to the usual, often already demanding drug list. Very difficult if protein- free food is not available. Expensive where supplements and protein-free foods are not supplied by the health care system	Scarce room for personalization; not flexible; Educational approach has also to be focused on compliance; has to be combined with a prescription approach (supplements).

#### Vegan-vegetarian diets

In the general population, veganism and vegetarianism are not only diets, they are integrated into a philosophy of life. This is a setting in which a mainstream approach (need to reduce proteins) finds a fertile ground in the growing attention to a “healthy” lifestyle and in a philosophy of life which ranges from respect for animals to avoiding diseases [91–95]. Hence, such diets are often listed along with CAMs in the treatment of diseases other than CKD. Overall, the protein content in vegan diets is lower than in omnivorous diets, and it is roughly 0.7 g/Kg/day. However, they are usually considered together with the “0.6” omnivorous diets on account of the lower absorption and bioavailability of vegetable proteins and of their lower effect on renal filtration [96–105].

Vegan-vegetarian diets are often discussed together since the distinction between them is relatively new. The term vegan was coined in 1944 by Donald Watson: “vegan” from the beginning and end of vegetarian “because veganism starts with vegetarianism and carries it through to its logical conclusion”. The two definitions were often used interchangeably in the past, however, these diets are different: vegan diets avoid any foods derived from an animal source, including eggs and dairy products [91–95]. The list of variants is long and includes various combinations such as ovo-vegetarian (eggs only), lacto-vegetarian (milk and dairy products), pescetarian or pesco-vegetarian (fish is allowed). As for vegan diets, honey is excluded by some, and allowed by others and the same is true for shellfish. In a world of processed food, vegans-vegetarians try to avoid all animal-derived components, such as sugars that are whitened with bone char, cheeses that use animal rennet, or gelatine from collagen. The attitude of “no harm” is extreme in fruitarianism, which is based upon fruit, nuts, seeds, and food gathered without “harming the plant” [94, 95, 106].

It is thus clear that, beyond the CKD context, the differences are not related to “food” but also to the “concept of food” and, more extensively, to the concept of life [91–95].

It is worth remembering a paper which signalled a new interest in LPDs in CKD patients which appeared about 30 years ago in the BMJ. It dealt with CKD in a group of Buddhist monks as compared with other subsets of Indian CKD patients. The very low protein content was indicated, together with the practice of meditation, as the putative cause of the very low progression rate in the monk population; at that time, the fact that Buddhist monks are vegetarians was not mentioned [107, 108]. Collaterally, we may also cite that prayer and meditation are presently considered the most widespread CAM worldwide [109, 110].

Regardless of the reason for making this choice, be it medical of personal, the preparation of vegan food is time consuming and attention must be paid to integrating different types of cereals, nuts and legumes into each meal to avoid nutritional deficits; however, provided deficits are avoided, these diets are safe in all phases of life, including pregnancy and lactation [111–114].

#### Supplemented vegan diets

Vegan-vegetarian diets are the basis for further integration into different schemes: very low protein (or “0.3”) diets are basically vegan and are supplemented by protein-free foods to supply energy, and by a mixture of aminoacids and ketoacids; a “0.6” simplified scheme has also been developed, with promising results also in elderly, diabetic or pregnant CKD patients [115–124]. Supplements are however “pills”; they may have an additive effect as scavengers of uraemic toxins, but they have to be prescribed, hence the educational and prescriptive approaches are combined. While different combinations of aminoacids and ketoacids were used in the past, in Europe two are available and are marketed by the same Company, the difference being the absence of tryptophan in the preparation that is available in Italy. The dose of the supplements is standardized in the “0.3” diet (1 pill every 5 Kg of body weight), while in the 0.6 diets a “compromise dose (1 pill/10 Kg) is chosen in an effort to find a balance between “too many pills” and the risk of malnutrition which is intrinsically linked to CKD and is enhanced by acidosis, depression, and possibly even by unbalanced diets [116, 125].

The role of supplements is different in “very low” and “low” protein diets: in the former they are needed to avoid protein malnutrition while in the latter, the addition of supplements allows to simplify the dietary scheme in previously omnivorous patients who are “forced” into a vegan regimen, or in patients who do not like protein-rich plant foods, namely legumes.

#### Omnivorous traditional diets

Hippocrates said that food is the first medicine.

Humankind has quickly shifted in the last century from relatively low food availability to a higher protein and energy supply. In many traditional diets, including Mediterranean diets or the extensively studied Okinawa diet (from one of the islands with the greatest longevity in the world), animal-derived food is a sort of “nutritional complement” on the basis of an otherwise vegan diet [126–134]. As a consequence, moderate protein restriction may be feasible in the context of several “traditional diets”. This issue is coming of age in a moment in which attention is being placed on healthy food and on consuming food that is processed minimally or not at all, which is possible only if it is produced in the surrounding area (i.e., “zero Km” food), thus implicitly supporting a return to a more traditional cuisine.

Adaptation may be difficult in settings where the baseline diet is poor in vegetables, fruits, cereals and legumes; however it should be kept in mind that animal-derived protein content has only recently risen in the “rich” world and that most traditional or “poor” cuisines have a relatively low protein intake [134–137]. Taking into account that CKD is mainly a disease of the elderly and that the sharp increase in protein intake occurred in most of the western world less than one century ago, designing tailored traditional diets in CKD may follow an “historical” educational approach in which patients are solicited to go back to their childhood to find adequate or adaptable dietary patterns.

#### 0.6- diets with protein-free food

Protein-free food is an idea that was “made in Italy” and conceived in an era of limited dialysis availability: “protein-free pasta” was invented in the sixties in a search for calorie-rich, protein-poor food [138].

In fact, other natural protein-free, energy-rich foods (tapioca, butter, sugar, fruits, vegetables) could be combined only in very monotonous diets which were extremely difficult to follow especially by patients who were already experiencing the deleterious effects of uraemia and acidosis on both their appetite and on gastrointestinal motility. The systematic use of protein-free food is limited to Italy where these products are available free of charge to CKD patients and where up to half of the protein intake derives from bread and pasta (12 g of protein per 100 g). Replacing these foods allows patients to reach the target protein intake without making any substantial changes in dietary habits [138–141]. In Italy, these diets are often well followed by elderly patients while, at least in our experience, younger patients prefer vegan approaches [142–145]. A prescription approach focused on consuming bread and pasta “from the pharmacy” is usually enough. Since however they are less palatable than what people are used to, education, aimed at understanding the reasons and goals of the LPD and at suggesting specific ways of cooking food to improve taste, represents an important strategy for improving adherence and coping.

### Mainstream, complementary medicine and CKD: where is the diet?

On Google, the term “mainstream medicine” retrieves over 3 million results and there are several different definitions among the first hits. Some of them are tautological and some are centred on “who” practices the medicine: “Medicine as practised by holders of M.D. or D.O. degrees and by their allied health professionals” [146]. Other definitions focus on the type of treatment “A system in which medical doctors and other healthcare professionals (…) treat symptoms and diseases using drugs, radiation, or surgery” [147]. Others are more complex and include an explanation of the pathogenesis of the diseases: a general term for conventional healthcare based on the “western model” of evidence-based practice for diagnosing and treating disease. Mainstream medicine assumes that all physiologic and pathological phenomena can be explained in concrete terms, and “best practice” is the final result of a stream of objective analyses. They begin with non-human model systems, evolve through blinded studies and a statistical analysis of the results, finally leading to guidelines to which doctors should adhere in order to achieve optimal patient outcomes [148]. In addition, they also include social elements: “The approach to healthcare as practised in developed nations is based on scientific data for diagnosing and treating disease; mainstream medicine assumes that all physiologic and pathological phenomena can be explained in concrete terms; the tools of mainstream medicine include non-human model systems, blinded studies, and statistical analysis to ensure reproducible results” [149].

Diet or nutritional approaches are never cited within these definitions of mainstream medicine. Conversely, on Medline plus, along with a general definition of Complementary and alternative medicines, CAM is the term for medical products and practices that are not part of standard care. Standard care is what medical doctors, doctors of osteopathy, and allied health professionals, such as nurses and physical therapists, practice. Dietary supplements are cited, while natural products are in first place in the classification of CAMs in the National Institute of health website [150, 151].

### Diet prescription in mainstream medicine and CAMs

The concept of diet may however be different in mainstream and complementary medicine: in the first case, it is usually based upon specific, measurable prescriptions, while in the second case it is more commonly part of a lifestyle, often with emphasis on disease prevention [7]. The limits are elusive, and no wonder, therefore, if a broad search strategy on Medline, combining the terms “Chronic kidney disease” and “alternative” or “complementary” or “allied” medicine retrieves, along with papers on CAMs, several studies on diets which are often defined as “alternative” to early dialysis, or “complementary” to pharmacological treatments. Thus, even if diet in CKD is not strictly considered a CAM, the terms currently employed for defining CAMs (complementary or alternative) identify many relevant recent papers on the dietary treatment of CKD [152–157].

The use of these terms is probably not only a semantic exercise: diet is often cited as an alternative to early dialysis or as a treatment that is complementary to ACE-inhibition or to other drug therapy (for instance, diuretics) [20, 158–161]. In this regard, a critical observation from the CAM point of view may offer some insights for a better understanding of the limits of applying “conventional” evidence-based medicine (EBM) analysis to low-protein diets in CKD, as well as into the possible role for a “whole system” analysis and for a narrative, personalized approach to maximize compliance (or, perhaps better stated as “concordance”) to low-protein diets in CKD patients [162–165].

### Personalised medicine, or RCTs? The lesson of the holistic, whole system approach

In the era of personalised medicine, we might wonder whether a randomised controlled trial is the best way to demonstrate the effects of changes in dietary habits and, more than this, if it is ethically sound to randomise such an intrusive lifestyle change [166–169].

In this regard, mainstream medicine should reflect on the lesson learned from CAMs and the focus might shift from proving the superiority of a single treatment (diet vs no diet; moderately- versus severely-restricted diets etc.) to an observational multidimensional “whole system” approach [7, 170–177].

A very interesting health technology assessment on the role of the preferences of professionals and patients in randomised controlled trials states that “All RCTs in which participants and/or professionals cannot be masked to treatment arms should attempt to estimate the participants’ preferences….” [178]). Current approaches may not be fit to highlight differences, and other study designs, in particular the new “partially randomized patient preference (PRPP) trial model”, which was developed for the analysis of CAMs, have already been applied to several fields of mainstream medicine [179–186].

No study is without pitfalls, and there are sound criticisms to virtually every study design [187]. However, according to an educational report in the BMJ: “when the effectiveness of the intervention depends on the subject’s active participation (…) a randomised trial may be inappropriate because the very act of random allocation may reduce the effectiveness of the intervention” [173].

If the focus on diets has to shift from issues regarding effectiveness to those of feasibility, then the implementation strategy should probably be personalized and should take into account the features of each patient [142]. This “narrative” approach may be seen as the other side of the moon as compared to a randomised trial approach. However, evidence-based medicine is fully compatible with narrative, individualized approaches provided that flexible, comprehensive and innovative study designs are chosen [188–190]. Paraphrasing a JAMA paper “Scientific reports (or RCTs) are genuinely dispassionate, characterless, and ahistorical. But their translation and dissemination (implementation in clinical practice) should not be” [191]. Dealing with personalized medicine, we should probably learn from patients’ preferences, and be happy with the fact that a treatment may have positive results, at least in experienced centres, in the absence of sound evidence on severe drawbacks and side effects. In this regard, the lessons learned from dialysis, which is chosen on the basis of the patients’ preference in the setting of an overall equivalence of side effects, may be extended to the diet, which is probably wrongly considered less intrusive in daily life than the choice of the different types of renal replacement therapy [69–71].

### What we did not discuss, but should not forget

Talking about diet, broadly defined on Wikipedia as “everything we eat”, is extremely complex and no review of a single issue, such as protein intake, can be exhaustive without touching upon other aspects, such as caloric intake, vitamins and oligo-elements, additives and contaminants. In particular, this is crucial in CKD where the presence of kidney disease impairs the activation of vitamin D, leading to a deficient absorption of calcium and to hyperphosphataemia; low-protein diets are usually deficient in vitamin B12, vitamin D, and iron. Conversely, hyper-vitaminosis may be a threat, in particular for vitamin D [192–194]. The toxicity of additive and trace elements, such as pesticides, has only recently been studied; negative effects are likely to be enhanced by reduced renal depuration [194–196]. We know less about individual elements, such as selenium, which is important in the oxidative pathway; furthermore, the network of saturated-unsaturated fatty acids is extremely complex and only partly known, and bicarbonate supplementation is still an open issue, while the discussion on all these items is beyond the scope of this review [197–199]. Again, a flexible holistic approach, which is more common in CAMs than in mainstream medicine, may be of interest. Allowing at least one (and in our experience up to three) free meals per week may help avoid deficits, generically speaking by enriching the diet, but the idea that “body wisdom” can help identify which foods the body needs is appealing [24, 200]. A holistic approach not only to the patient, whose diet is an important part of daily life, but also to the diet as a whole and not only as a “reduction of” may reduce the risk of focusing on single elements, and thus losing sight of the whole problem.

### Tips for counselling

There is probably at present no best low-protein diet, but, in the absence of contraindications, the best approach is probably to offer the patients a choice of diets with different options and protein content, bearing in mind that the most important issue is not the optimal prescription, but the optimal compliance and target achievement (Table 2).

In this regard, the prescriptive approach that deals with low protein diets as it does with drug therapies may be combined with the educational approach that is typical of CAMs by integrating dietary habits into daily life [201–203].

Patients should be aware that there is a high degree of uncertainty with regard to the choice of their dietary treatment and that its aim is to reduce the signs, symptoms and complications of renal failure and then to delay the need for dialysis. Furthermore, even if this is beyond the scope of this review, they should be warned that a low-protein diet is not necessarily a healthy diet, and that several other factors, such as type of renal disease, type of proteins, salt and phosphate intake, use of canned and processed food, and effective energy intake can modulate the risks and the clinical success [194–196].

### Suggestions for research

Long-term interventions that require a profound change in everyday life are difficult to assess, at least with the current study strategies. Clinical research needs long-term observations and long term follow-up, whenever possible even after the start of dialysis, to check for carry-over effects and for attrition biases. Since the report and publication biases suggest that only experienced centres with sound, positive data should publish their series and studies, efforts should be made to build large multi-centre series involving centres with different types of experience on low-protein diets. Multi-centre studies should take into account the important cultural differences in eating habits and cuisine which are often present even within the same country. Both “per protocol” and “intention to treat” analyses may be relevant in chronic interventions and both should probably be analysed [203, 204].

Since it is somewhere between a mainstream prescription and a complementary therapy, the study of diet in chronic kidney disease should merge the suggestions of both approaches. Integration among various study designs that take into account patients’ preferences (observational, randomised, partially randomised, etc.) is more promising than focussing on a single type of analysis, be it randomised or observational.

## Conclusions

There is no ideal low-protein diet for all CKD patients.

However, on the basis of the present evidence which shows an advantage in terms of dialysis-free follow-up, and a lack of advantage (or even a disadvantage) in early dialysis start, all CKD patients should be offered a “good diet”, or better, an option among various “good diets” since the best one is the one that is truly followed by the patient. The methodological flaws and limitations of the present evidence are probably also linked to the fact that “traditional” RCTs are based upon an assumption of equal preferences that is not applicable to low-protein diets.

A diet is not a drug.

Diet integration into daily life is highly dependent upon preferences and personal choices, and in this regard, the experience of CAMs may be important for integrating this potentially precious tool into the life of our patients, shifting our efforts from standardization to adaptation, as per the lesson of narrative medicine.

These approaches do not clash with classic, evidence-based medicine; on the contrary, they need solid EBM support, integrating the conventional, more rigid study designs, such as RCTs, with new ones, such as whole-systems approaches or partially randomised patient preference trials.
